# Study on the Compressive Stress Retention in Quenched Cam of 100Cr6 Steel Based on Coupled Thermomechanical and Metallurgical Modeling

**DOI:** 10.3390/ma14205912

**Published:** 2021-10-09

**Authors:** Jianbin Chen, Zhidong Zuo, Songze Zhou, Xiaofeng Wang, Yonglong Chen, Guoping Ling

**Affiliations:** 1 School of Mechanical Engineering and Mechanics, Ningbo University, Ningbo 315211, China; zuozhidong0919@163.com (Z.Z.); m17835395484@163.com (S.Z.); wangxiaofeng@nbu.edu.cn (X.W.); 2 SLPT State-Level Enterprise Engineering Center, Ningbo Shenglong Group Co., Ltd., Ningbo 315105, China; yl.chen@sheng-long.com; 3 School of Materials Science and Engineering, Zhejiang University, Hangzhou 310014, China; linggp@zju.edu.cn

**Keywords:** assembled cam, 100Cr6 steel, quenching, phase transformation, compressive stress retention

## Abstract

The assembled camshaft has obvious advantages in material optimization and flexible manufacturing. As the most important surface modification technique, the heat treatment process is utilized in this work to promote the desired compressive residual stress on the near-surface of the 100Cr6 steel assembled cam. The Johnson-Mehl-Avrami equation and Koistinen-Marbuger law are integrated into the ABAQUS software via user subroutines to simulate the evolution of diffusional transformation and diffusionless transformation, respectively. The linear mixture law is used for describing the coupled thermomechanical and metallurgical behaviors in the quenching of steel cam. The influences of various quenchants and the probable maximum phase volume fractions on surface residual stress or hardness are analyzed. Results show that a greater amount of martensite volume fraction and a slower martensitic transformation rate are beneficial for the compressive stress retention. Compared with the conventional quenching oil, the fast oil quenched cam surface has higher final compressive stress and hardness.

## 1. Introduction

In the traditional automobile industry, the power performance and fuel economy of vehicle are greatly affected by the engine performance. As one of the key components in automobile engine, the camshaft is used to control the continuous open and close of valves or fuel injection pumps for the normal working of the engine [1]. Therefore, both the kinematic and dynamic characteristics have been widely taken into consideration in the design and optimization of the complex cam profiles to meet the overall engine performance [2,3,4]. In early stages, the conventional integral camshaft was mainly manufactured by casting or forging with a limited choice of material and processing. For satisfying the new requirement of manufacturing accuracy, the assembled camshaft by the interference fit has been gradually adopted in the vehicle engine due to its tremendous advantages in material selection and flexible design and manufacturing [5]. On the other hand, due to the working conditions with cyclic stresses and impact loads, the failure behaviors such as wear and crack occur frequently on the cam surface. Different types of materials and heat treatment processes have been introduced for improving the strength and wear resistance of the cam. For example, 100Cr6 is a typical surface-hardened steel widely used in the assembled camshaft of engine parts with a radial knurling connection for its excellent wear resistance and high hardness after martensite surface hardening. Since the microstructural phase in the steel changes at specific heating or cooling rates within a certain holding time, the thermal stress and structural stress are generally introduced in the sample simultaneously during martensite surface hardening [6,7]. Finally, the coupled temperature, phase transformation, and stress problem turn out to be remarkable to control the distortion and residual stress on the cam surface.

In fact, three major processing techniques including heat treatment, press fitting, and grinding are generally involved during the production of an assembled camshaft. As a consequence, it has become a commonplace that cracks are prone to initiate at the cam surface, especially around the transition region for a typical cam profile composed of three sections, i.e., the base circle section, the buffer section, and the working section. In order to improve the surface integrity of the component, the mechanisms of the above three major processing techniques have attracted much attention by researchers. Zhang et al. [8] investigated the joint mechanism of assembled camshaft and predicted the connection strength for a radial knurling connection. Shah et al. [9] investigated the grinding induced phase transformation and residual stresses of AISI 52100 steel. Madopothula et al. [10] studied the effect of rapid quenching on the white layer formation mechanism in grinding of AISI 52100 steel. Hunkel et al. [11] utilized the dilatometer to study the tempering effects of athermal martensite on the strain development behavior during quenching and reheating of 52100 steel. Three tempering stages including the time-dependent formation of transition carbides, the loss of tetragonality triggered by transition carbides, and the retained austenite transformation caused by austenite stabilization were analyzed in detail. Sidoroff-Coicaud et al. [12] experimentally investigated the dimensional stability of 52100 steel under different heat treatment conditions. It was shown that both the retained austenite volume fraction and the location of carbon after quenching could lead to an expansion in service. Li et al. [13] carried out orthogonal and single-factor experiments to investigate the residual stresses during the heat treatment and grinding processes of GCr15 steel cam. Results showed that the priority of the influence factors on the compressive residual stresses was grinding speed, quenching power, and tempering temperature in sequence. In addition, an extremely high temperature is likely to generate in the grinding zone during material removal. Therefore, the solid-state phase transformations may happen again due to the second quenching. It is remarkable that several surface cracks are found to be initiated before press fitting. Therefore, the heat treatment is always taken as the most important processing to balance the residual stress and the mechanical properties during the assembled camshaft production.

Compared with the traditional practical experience method, the numerical simulation has been developed as a useful tool to predict the thermomechanical and metallurgical behaviors caused by the heat treatment for about 60 years [14]. Li et al. [15] discussed the testing and data required to model the phase transformation kinetics for the heat treatment of steel parts. Denis et al. [16,17] presented a mathematical model for the calculation of phase transformations in steels during rapid heating and cooling based on the Johnson-Mehl-Avrami law, as well as the additivity rule with consideration of local carbon content and austenite grain size. Houghton et al. [18] conducted a series of heat treatment experiments in 52100 steel and the Avrami equation was adopted to determine the isothermal kinetics of the austenite to bainite transformation. Esfahani et al. [19] proposed a coupling phase transformation numerical model to investigate the microstructure evolution and residual stress in quenched steel. The stress state and hardness affected by ferrite formation and pearlite phase transformation have been analyzed. Simsir [20] used the stressed dilatometry technique to determine the transformation induced plasticity (TRIP) parameter of 52100 steel and investigated the effect of applied stress on phase transformations. Yaakoubi et al. [21,22] developed the user subroutines via the ABAQUS software to analyze the metallurgical and mechanical behaviors of steels in the heat treatment such as phase fractions, hardness, and stress genesis. Lingamanaik et al. [23] combined the heat treatment software DANTE with ABAQUS to promote favourable compressive residual stresses in steel by altering the quenching parameters such as heat transfer coefficients, quenching duration, and quenching locations.

The aim of this paper is to investigate the evolution of phase transformations and stress in the quenched engine cam manufactured by the hardened steel 100Cr6. The Johnson-Mehl-Avrami equation and Koistinen-Marbuger law are integrated into ABAQUS through user subroutines to predict the diffusional transformation and diffusionless transformation, respectively. The effects of different quenchants and the probable maximum phase volume fractions on phase transformations are analyzed in order to promote the desired compressive residual stress on the near-surface of the assembled cam.

## 2. Materials and Methods

In the heat treatment process, the complicated interactions among the heat transfer, phase transformation, and mechanical behavior affect the mechanical properties significantly. To perform the quantitative prediction of phase fractions and residual stress during numerical simulation for the heat treatment of assembled cam, the thermodynamics theory and phase transformation kinetics theory are summarized as follows.

### 2.1. Thermal Modeling

The temperature distribution over the thickness of the assembled cam can be obtained by solving the heat conduction problem, as described in Equation (1) [14].

(1) $$c\rho \frac{\partial T}{\partial t}=\mathrm{div}\left(\lambda \mathrm{grad}T\right)+\dot{q}$$

where c is the specific heat, ρ is the density, T is the temperature, λ is the thermal conductivity, $\dot{q}$ is the rate of latent heat caused by phase change, and both div and grad represent the divergence and gradient operator, respectively.

According to the additivity rule, the rate of latent heat is expressed as Equation (2) [16].

(2) $$\dot{q}=\sum_{k=1}^{5}\Delta H_{k}\frac{df_{k}\left(r,t\right)}{dt}$$

where $\Delta H_{k}$ represents the enthalpy change when the phase transforms into the constituent *k*, $f_{k}$ is the phase fraction of the constituent *k* (*k* = l austenite, *k* = 2 ferrite, *k* = 3 pearlite, *k* = 4 bainite, *k* = 5 martensite).

With the third boundary condition in Equation (3) [14], the temperature can be calculated.

(3) $${\left.-\lambda \frac{\partial T}{\partial n}\right|}_{S}=h\left(T-T_{f}\right)$$

where h is the convection heat transfer coefficient between the cam and quenchant, $T_{f}$ is the final temperature of the cam surface. According to Wang [24] and Lee [25] et al., the convection coefficients for different quenching media can be successfully calculated by the inverse algorithm using the measured temperatures of quenched steel. They pointed out that the temperature dependent convection coefficient was mainly determined by the inherent cooling characteristic of the material, especially the latent heat generated by phase transformation.

### 2.2. Metallurgical Modeling

In the quenching of 100Cr6 steel, two kinds of transformations may occur depending on the underlying mechanism. One is called diffusional transformation such as ferritic, pearlitic, and bainitic transformations. The other is called diffusionless transformation, such as martensitic transformation. The phase transformation kinetics must be known to calculate the phase fractions, in order that the interactions among the temperature, stress, and phase transformation can be established through the additivity rule associated with phase fractions.

For diffusional transformation, the Johnson-Mehl-Avrami formula, as shown in Equation (4) [14], is utilized to describe the nucleation and growth of a new phase.

(4) $$f_{k}=f_{k}^{\max}\left(1-e^{-b_{k}t^{n_{k}}}\right)$$

where $f_{k}^{\max}$ is the maximum phase fraction of phase *k*, $b_{k}$ and $n_{k}$ are temperature dependent phase transformation kinetics parameters, which can be obtained from the time temperature transformation (TTT) curves during isothermal transformation.

Additionally, the martensitic transformation is usually considered to be time independent. The Koistinen-Marburger relation given in Equation (5) [14] is introduced to describe the progress of diffusionless transformation.

(5) $$f_{m}=f_{m}^{\max}\left[1-e^{-\gamma \left(M_{s}-T\right)}\right]$$

where $f_{m}^{\max}$ denotes the maximum volume fraction of martensite, which can be obtained from the continuous cooling transformation (CCT) diagram, $M_{s}$ is the start temperature of martensitic transformation, γ is a constant with its value close to 0.011 K^−1^ for most of the steels.

### 2.3. Mechanical Modeling

During the heat treatment, a material undergoes thermal loading and microstructure development. To be able to describe the mechanical response, the classical plasticity flow rule associated with von Mises yield criterion and isotropic hardening is used for stress calculation. The total strain increment $d\varepsilon_{ij}$ can be decomposed into different individual strain increments, as shown in Equation (6) [21].

(6) $$d\varepsilon_{ij}=d\varepsilon_{ij}^{e}+d\varepsilon_{ij}^{p}+d\varepsilon_{ij}^{th}+d\varepsilon_{ij}^{tr}+d\varepsilon_{ij}^{pt}$$

where $d\varepsilon_{ij}^{e}$ is the elastic strain increment related to the stress increment by the Hooke’s law, $d\varepsilon_{ij}^{p}$ is the plastic strain increment calculated by combining the classical plasticity theory with the associated hardening rules, $d\varepsilon_{ij}^{th}$ is the thermal strain increment depending on the temperature and the expansion of the different phases, $d\varepsilon_{ij}^{tr}$ is the phase transformation strain increment related to the volumetric expansion of phase transformation, $d\varepsilon_{ij}^{pt}$ is the transformation induced plasticity strain increment, which can be expressed as a function of the deviatoric stress.

The thermal strain and transformation strain are phase dependent and calculated as Equations (7) and (8) [16], respectively.

(7) $$d\varepsilon_{ij}^{th}=\sum_{k=1}^{5}f_{k}\alpha_{k}\left(T\right)dT$$

(8) $$d\varepsilon_{ij}^{tr}=\sum_{k=2}^{5}f_{k}d\varepsilon_{k}^{\Delta V}$$

where $\alpha_{k}$ is the thermal expansion coefficient related to the constituent *k*, $\varepsilon_{k}^{\Delta V}$ is the volumetric expansion when the austenite transforms into the constituent *k*.

According to Rohde et al. [14], the temperature dependent thermal expansion $\alpha^{*}\left(T\right)$ can be expressed as a function of the original density $\rho_{0}$ and the density ρ at the present temperature ($\alpha^{*}\left(T\right)=\left[{\left(\frac{\rho_{0}}{\rho }\right)}^{\frac{1}{3}}-1\right]\frac{1}{T}$). In addition, the thermal and transformation strain given in Equations (7) and (8) can be combined as $\varepsilon_{ij}^{th}+\varepsilon_{ij}^{tr}=T\alpha^{*}\left(T\right)$ when using a standard FEM program. For convenience, the latent heat, thermal expansion coefficients, and volumetric expansions caused by the phase transformation during the heat treatment of 100Cr6 steel are supposed to be temperature independent. In addition, they are summarized in Table 1 based on the means of dilatometric tests under various temperatures, performed by Denis et al. [16,17] and Yaakoubi et al. [21]. It is remarkable that the coupled thermomechanical and metallurgical behaviors of steel have been successfully predicted by Denis et al. [16] and Yaakoubi et al. [20], based on the given mean thermal expansion coefficients for multiphase materials.

### 2.4. Finite Element Simulation Modeling

The numerical simulation for the heat treatment of the hollow assembled cam is performed using the commercial finite element software ABAQUS. A brief description with reference to the establishment of the geometry, thermo-mechanical properties, constraints and loading conditions for heat treatment finite element simulation is presented as follows.

**Figure 1:**
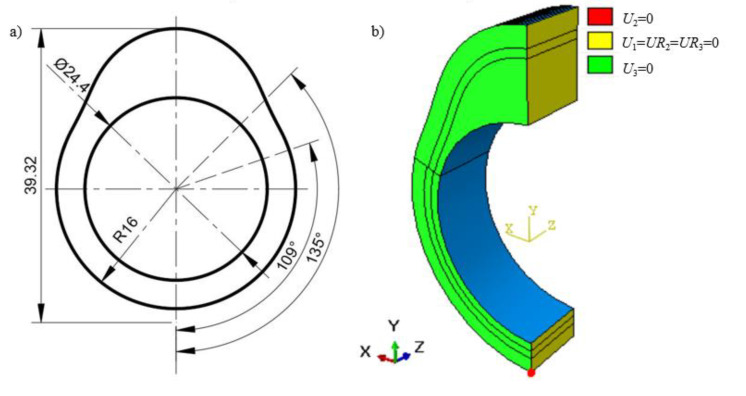
Model of the hollow assembled cam (**a**) dimensional drawing and (**b**) boundary condition.

**Figure 2:**
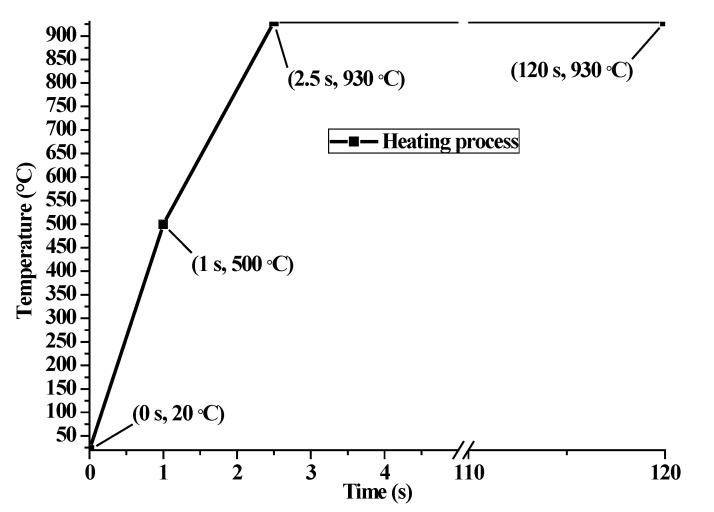
Temperature amplitude of the heating process.

**Figure 3:**
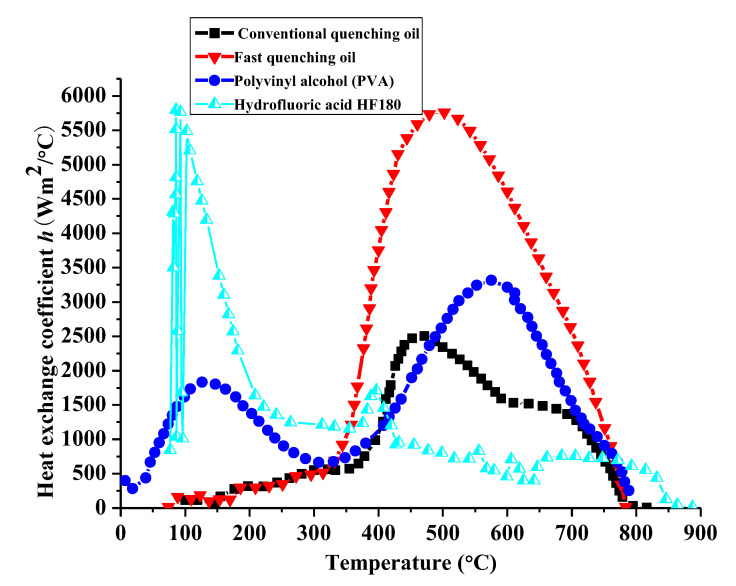
Variations of convection coefficient with temperature for the GCr15 steel in different quenchants.

The profile of the assembled cam in this paper is designed to be symmetrical, as shown in Figure 1a. The thickness of the cam is *h*_z_ = 8 mm. During the heat treatment, the material on all the cam surfaces can expand and contract freely except the bearing surface. For the convenience of calculation, a three-dimensional axisymmetric finite element model is established to analyze the heat treating process of the cam based on the symmetry in mechanics and shape, as given in Figure 1b. The symmetric surface is restrained with symmetric constraints on the X-axis. The freedom degree along the axial direction *U*_z_ of the bottom surface and the freedom degree along the Y-axis of a point *Q* are restrained, as well. The thermal loading presented in Figure 2 is applied on the cam profile surface to simulate the heating process. Four different heat transfer coefficients referring to Wang [24] are defined at the free surface of the cam to investigate the heat exchange by convection between the specimen and quenchant, as shown in Figure 3. The element type C3D8RT and the element shape Hex are used in the coupled thermal and mechanical model analysis. In addition, a long enough cooling time of 1200 s is recommended to acquire a precise phase volume fraction. Moreover, the residual stress at the end of quenching as the finish temperature of martensitic transformation is supposed to be around room temperature.

**Figure 4:**
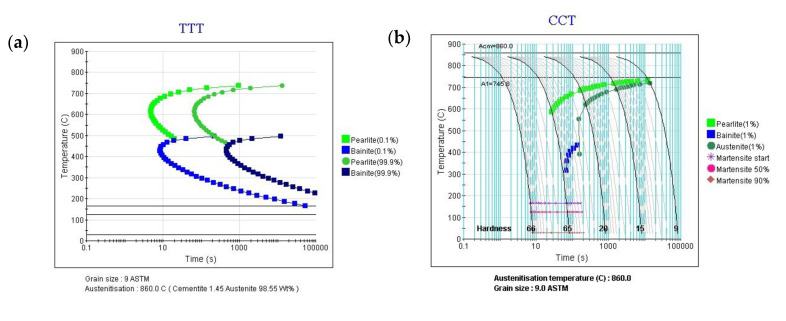
Phase transformation curves of 100Cr6 steel for (**a**) time-temperature-transformation and (**b**) continuous-cooling-transformation.

The phase dependent expansion, heat generation, and plastic parameters of the heat-treated 100Cr6 steel are defined through the user subroutines UEXPAN (User subroutine to define incremental thermal expansion), HETVAL (Internal heat generation), and UHARD (Define hardening parameters), respectively. In addition, the field variables associated with the evolution of material properties are defined by the user subroutine USDFLD (User defined field) and stored in solution-dependent state variables via Depvar. The transformation temperatures for the 100Cr6 steel are *A*_c1_ = 75 °C, *A*_c3_ = 860 °C, *P*_s_ = 700 °C, *B*_s_ = 500 °C, *M*_s_ = 170 °C, and *M*_f_ = 30 °C. It is worth mentioning that the linear mixture law is used for evaluating the thermal and mechanical properties of multiphase material. Although the temperature dependent mechanical and thermal properties for the 52100 steel have been given by Shah et al. [9], some of the material properties are supposed to be phase and temperature independent in the numerical simulation for convenience, as shown in Table 2. Moreover, the chemical constituents of 100Cr6 steel, presented in Table 3, are submitted to the thermodynamic software JMatPro 7.0, in order that the isothermal transformation and continuous cooling transformation diagrams of the material, as presented in Figure 4, can be easily generated.

### 2.5. Experiment

The cam used in our study is provided by Daemyung Precision Machinery (Zhangjiagang) Co., Ltd., Zhangjiagang, China. The primary processing techniques of cam blank are forging, spheroidizing annealing, and shot-blasting. The forging is carried out based on the use of a Hatebur AMP 20 machine made by Hatebur Swiss Precision AG from Brugg, Switzerland. The temperature and time for bright spheroidizing annealing are 785 °C and 16 h, respectively. After annealing, the cam is processed by shot-blasting for 35 min. Based on the provided cam blank, the cam sample is then surface hardened by the high frequency induction quenching equipment. The electrical power, supply frequency, and heating time are set as 108 kW, 28 kHz, and 1.98 s, respectively. The heated part is rapidly cooled by the quenching oil provided in Table 4. The heat treatment oil classification adopted in this paper refers to the Petrochemical Industry Standard of the People’s Republic of China (SH/T 0564-93). The residual stress and retained austenite content tests are carried out on the X3000 G2/G2R stress measuring instrument from Stresstech Oy, Jyväskylä, Finland. The details about XRD measurements are summarized as follows: Material-Ferrite, miller indices—(211), 2θ angle—156.4°, Tube type—Cr, Exposure time—10.0 s, No. of tilts—4, and the tilt angle from −45 to 45°. In addition, the HR-150A Rockwell hardness and Vickers hardness tester provided by Laizhou Huayin Testing Instrument Co., Ltd. (Yantai, China) are used for determining the surface hardness and hardened layer thickness of the heat-treated cam.

## 3. Results and Discussion

### 3.1. Effect of Convection Coefficients on Hoop Residual Stress

**Figure 5:**
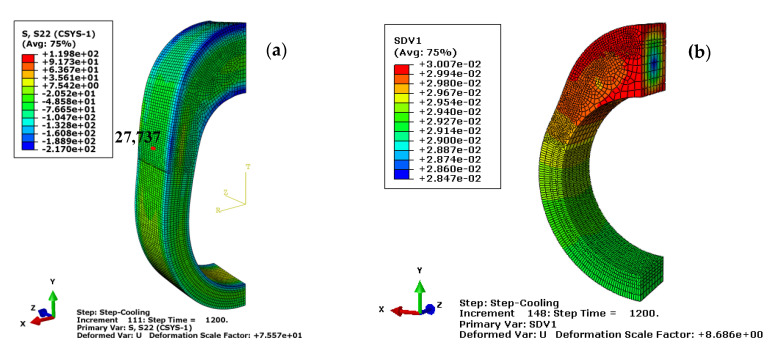
Multi-physics of the quenched cam (**a**) hoop stress and (**b**) retained austenite content.

The cooling rate during quenching significantly influences the final microstructures and mechanical properties of steel. The four quenchants with different convection coefficients, presented in Figure 3, are utilized to analyze the coupling effects among the temperature, microstructure, and stress. Since the different temperature histories appear on the complicated cam surface profile, a weak area with an unfavorable residual stress state often occurs around the buffer section during induction hardening. Therefore, the evolution of multi-physics field of the No. 27,737 element in the transition zone, as shown in Figure 5a, is traced to better understand the interaction between the microstructure and mechanical property. In the following, the hoop stress defined in the cylindrical coordinate system O-RTZ will be analyzed based on the crack nucleation and propagation in the production practice. Usually, the hoop stress is represented by the forces acting towards the circumference perpendicular to the length of the cylinder.

**Figure 6:**
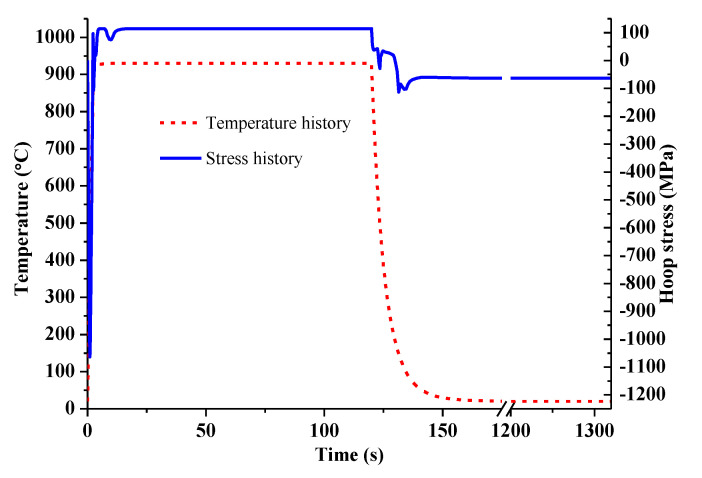
Stress history and temperature history on the specified element.

The stress history and temperature history on the cam surface during the whole heat treatment simulation process is shown in Figure 6. It can be seen that a large compressive surface stress occurs at the start of the heating moment as the volume expansion on the surface is suppressed by the inner layer. As the heat transfer continues, the compressive stress is rapidly released. When the surface temperature is above the complete austenitizing temperature, the tensile surface stress suddenly transforms into compressive stress and gradually releases afterwards with the temperature and microstructure homogenization. When the cooling step starts, the surface temperature drops sharply and the surface stress transforms from compressive to tensile, due to the fact that the volume shrinkage is restrained by the inner layer. As the temperature continues to drop, the volume expansion occurs again due to the diffusive and non-diffusive transformations. As a result, the structural stress plays a dominant role in the final surface stress state. The final compressive residual stress on the cam surface around the buffer section is −63.3 MPa. In addition, the predicted retained austenite content is around 3% at the end of the quenching process as shown in Figure 5b.

To verify the correctness of the finite element model, a series of surface residual stress tests are performed by the experiment equipment. Since the heat treated sample has a working allowance of 0.5 mm, a pre-etching is conducted before the stress test with the corrosion depth around the working allowance. For each group of the cam sample, the residual stress is measured twice for the consideration of equipment stability. Thirty sets of residual stress test results and several sampling results of retained austenite contents are listed in Table 5. The average stress value for the experimental test is −67.8 MPa and has a good agreement with the simulation result. In addition, the predicted value of retained austenite content in Figure 5b falls well in the interval of experimental results (2.9–6.6%).

**Figure 7:**
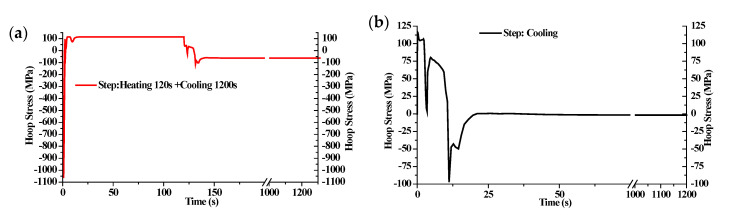
Stress history during quenching (**a**) with and (**b**) without considering the heating process.

In the following, the heating step will be neglected to improve the calculative efficiency when analyzing the influences of different quenchants on mechanical properties. Firstly, the stress histories during the heat treatment with and without the heating step on the specified element are compared with each other, as shown in Figure 7. It implies that the stress state tends to be compressive when the phase transformation suddenly happens during the cooling step and has a gradual release, since the temperature and microstructure fields become more uniform. The final surface residual stress without considering the heating step is −1.7 MPa. In other words, the real compressive residual stress after quenching is much higher than the predicted stress without considering the heating process.

**Figure 8:**
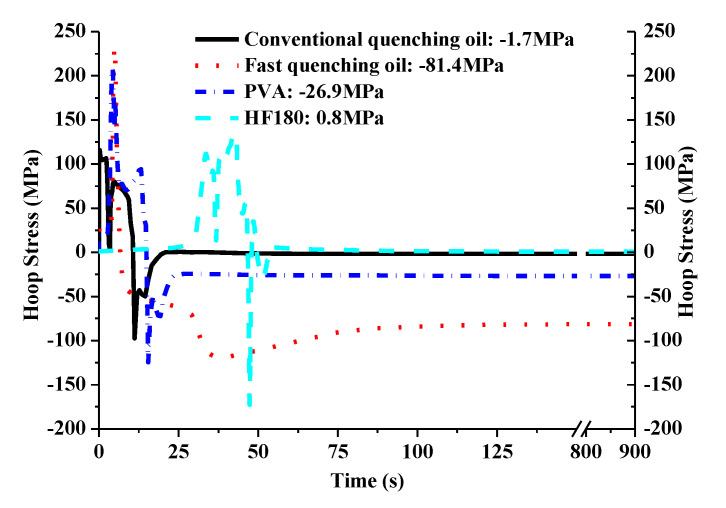
Surface stress of the specified element under various quenchants.

**Figure 9:**
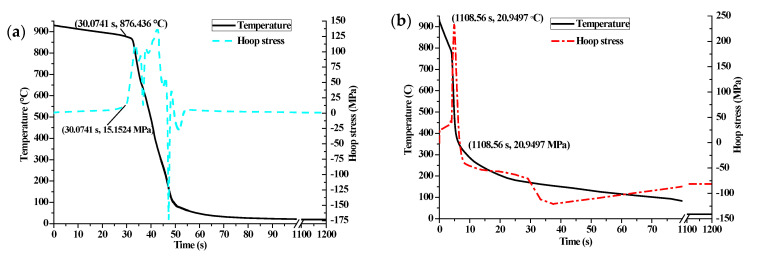
Temperature and stress comparison between (**a**) HF180 and (**b**) fast oil quenching.

**Figure 10:**
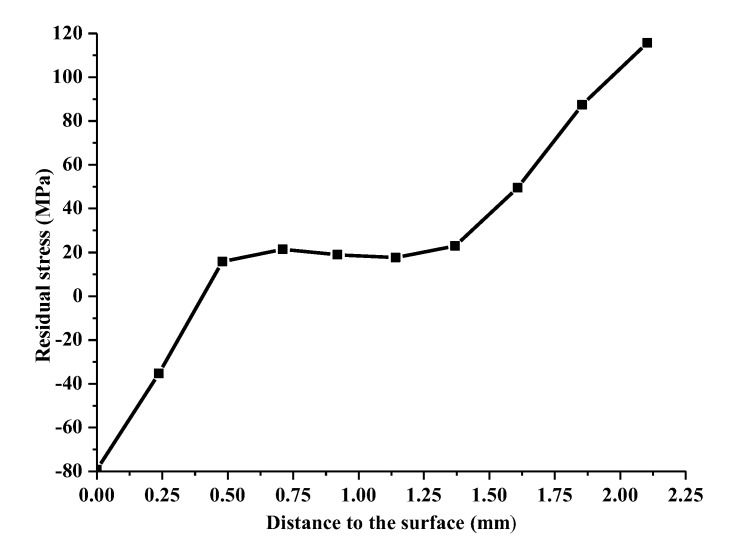
Residual stress depth profile for fast oil quenching of assembled cam.

The influence of different quenchants on the surface stress is shown in Figure 8. It can be seen that the fast quenching oil has the maximum rising rate of surface tensile stress at the beginning of the quenching process, due to the large heat exchange coefficient gradient at high temperature zone. Conversely, the surface tensile stress of the hydrofluoric acid HF180 quenched cam increases most slowly. As can be seen from Figure 3, the heat transfer coefficient of the HF180 quenchant is very small at high temperature above 876 °C. Specially, a default heat transfer coefficient corresponding to the sink temperature will be used for calculation when the surface temperature is beyond the given temperature range in Figure 3. Therefore, the stress does not rise rapidly until the temperature is lower than 876 °C, as shown in Figure 9a. Similarly, the surface stress for fast oil quenched cam in Figure 9b rises rapidly when the temperature is lower than the maximum temperature (778 °C) given in Figure 3. During the cooling process, the volume change caused by the phase transformation and nonuniform temperature determines the stress history together. The occurrence of martensitic transformation is beneficial to obtain a large compressive surface stress. In addition, a slow change of heat exchange and phase transformation in the low temperature zone is highly suggested to keep the compressive stress in the final state. As a result, the residual compressive stress for the fast quenching oil and polyvinyl alcohol (PVA) solution are −81.4 MPa and −26.9 MPa, respectively. The final surface stress state for the HF180 quenched cam is tensile with a value of 0.8 MPa. What is more, it can be seen in Figure 10 that the residual stress for fast oil quenched cam shifts from compressive to tensile along the depth direction. Therefore, the surface stress draws much attention to study the compressive stress retention. The reason for a flat region of residual stress profile below the surface layer in Figure 10 is mostly owing to the large thickness around the buffer section.

**Figure 11:**
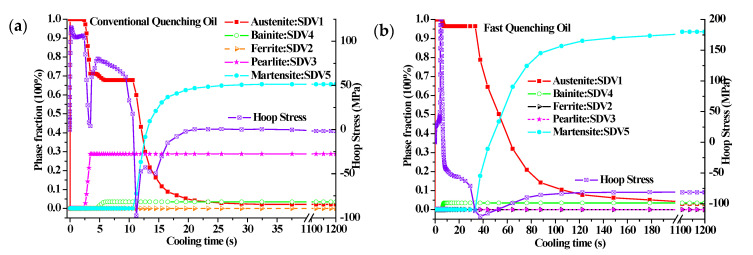
Relationship between the phase transformation and hoop stress under (**a**) conventional and (**b**) fast quenching oil.

**Figure 12:**
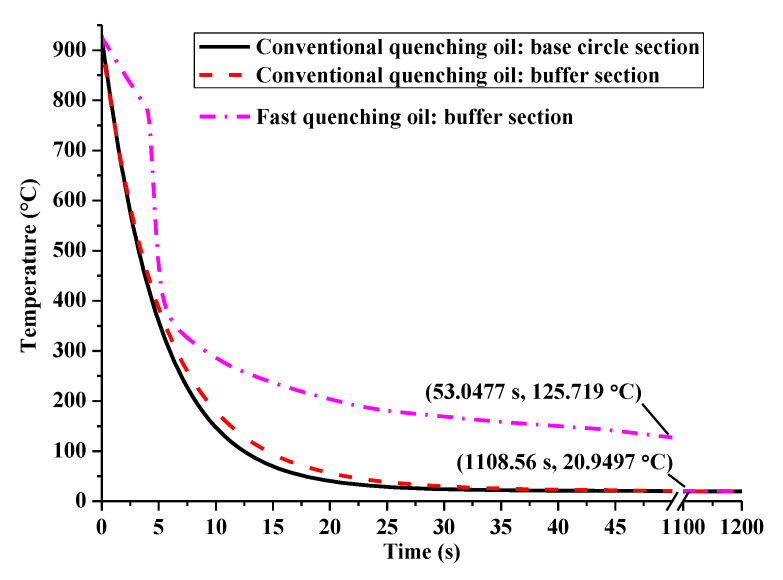
Temperature history for the two oil quenching media.

Based on the data in Figure 3, there is an obviously different trend of heat transfer coefficient between the PVA solution and quenching oil below the start temperature of martensitic transformation. However, the heat transfer coefficient for conventional quenching oil and fast quenching oil are in the same temperature range and have the unique peak value with various amplitudes around a certain temperature. The above mentioned features of the heat transfer coefficient make it easy to investigate the different evolution of phase transformations for the two quenching media. To better explain the formation mechanism of residual compressive stress, the phase transformation evolution under the conventional and the fast quenching oil are detailed, as compared with each other in Figure 11. Compared with the verified finite element model in Figure 7a, the only difference in Figure 11 is the negligence of heating process, which makes the prediction of phase transformation evolution, provided in Figure 11, still credible. It can be clearly seen that the stress tends to be compressive once a phase change occurs, and the maximum compressive stress usually happens at the start of martensitic transformation. Compared with the conventional quenching oil, the maximum compressive stress and the maximum martensite volume fraction of the fast oil quenched cam are larger. Moreover, though the conventional oil quenched cam has a maximum compressive stress of −97.3 MPa, the final residual stress is only −1.7 MPa due to the rapid stress relief caused by microstructure homogenization. Therefore, a slow martensitic transformation rate is favorable to obtain a quenching compressive stress eventually as, for example, the fast quenching oil shown in Figure 11b. In addition, the time period for phase transformation is greatly affected by the temperature history. It can be seen from Figure 12 that the surface temperature dropping rate for fast quenching oil is larger than the conventional quenching oil in the diffusional transformation period, while a smaller value in the diffusionless transformation zone is achieved. Especially, the heat transfer coefficient for conventional quenching oil is much lower than the fast quenching oil in the temperature range of perlite transformation (500–700 °C), in order that a longer phase change time is available for a large amount of perlite during conventional quenching oil of steel, as shown in Figure 11a. In addition, Figure 12 shows that the buffer section of the assembled cam has a slower temperature dropping than the base circle section. It is remarkable that the break region of temperature history from 50 to 1100 s has been shown in Figure 12 for an easy comparison.

**Figure 13:**
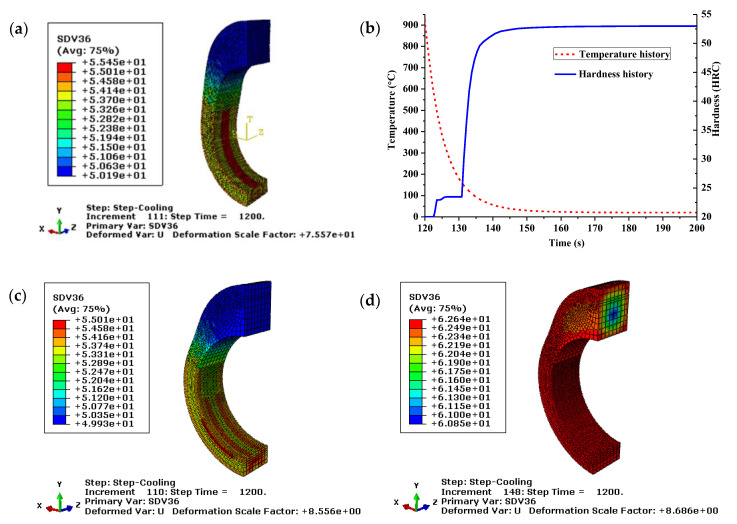
Hardness of the conventional (**a**–**c**) and fast (**d**) oil quenched cam with (**a**,**b**) and without (**c**,**d**) considering the heating process.

The hardness contours for different oil quenched cams are shown in Figure 13. It can be seen from Figure 13a,c that the final hardness of conventional oil quenched cam with and without considering the heating process are 50.19–55.45 and 49.93–55.01 HRC, respectively. The little difference between them indicates that the heating process can be ignored in order to improve the computational efficiency for the current problem. The hardness history in Figure 13b shows that the surface hardness increases gradually with the phase transformation during the quenching process. Especially, the hardness increases rapidly when the temperature is lower than the start temperature of martensitic transformation, due to the formation of high hardness phase during the non-diffusive transformation. Compared with the conventional oil quenched cam, the fast oil quenched cam in Figure 13d has a higher hardness value of 60.85–62.64 HRC. Since the quenching part is generally followed by a tempering process to obtain a good comprehensive mechanical property, the hardness and brittleness will be further decreased. Therefore, the fast quenching oil is recommended to ensure a good wearability with the hardness over 55 HRC. The surface hardness and microhardness distribution on the heat-treated sample are experimentally measured by the Rockwell and Vickers hardness tester for verification and the results are given in Table 6, Table 7 and Table 8. The mean hardness after quenching and tempering are 63.23 and 60.36 HRC, respectively. In addition, the hardened layer thickness is around 2.0 mm. Both the surface hardness and hardened layer thickness fall within the technical standard.

### 3.2. Effect of Different Maximum Phase Volume Fractions on Hoop Residual Stress

**Figure 14:**
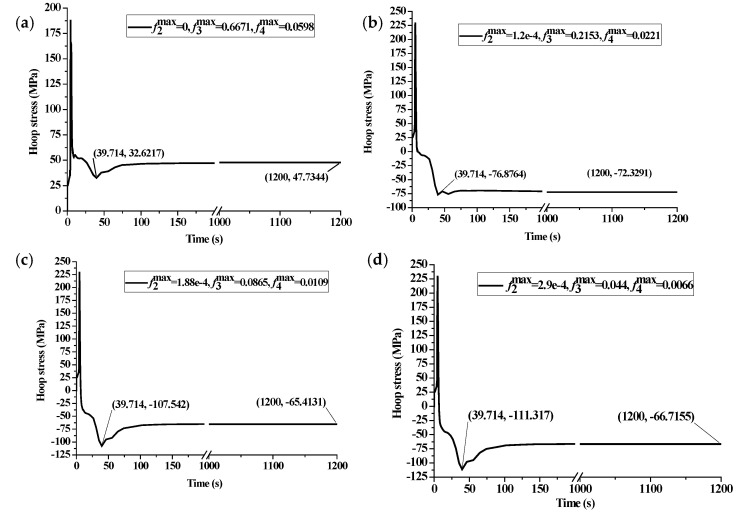
Stress history at various combinations of the probable maximum phase volume fraction. (**a**) $f_{2}^{\max}=0,f_{3}^{\max}=0.6671,f_{4}^{\max}=0.0598$; (**b**) $f_{2}^{\max}=1.2e^{-4},f_{3}^{\max}=0.2153,f_{4}^{\max}=0.0221$; (**c**) $f_{2}^{\max}=1.88e^{-4},f_{3}^{\max}=0.0865,f_{4}^{\max}=0.0109$; (**d**) $f_{2}^{\max}=2.9e^{-4},f_{3}^{\max}=0.044,f_{4}^{\max}=0.0066$.

According to the continuous cooling transformation diagrams in Figure 4b, several combinations of the probable maximum transformation fraction for each phase under certain cooling rates are given to investigate the influence of phases on surface stress. It can be seen from the surface stress history in Figure 14a that the residual compressive stress cannot be obtained when the final microstructure after quenching is pearlite in the majority ($f_{3}^{\max}=0.6671$). With the decrease of both pearlite and bainite maximum volume fractions, a larger maximum compressive stress can be obtained at the start of martensite transformation (t=39.714 s). It means that a certain amount of martensite volume fraction after quenching is the necessary condition for compressive surface stress. However, a large maximum compressive stress is not necessary to keep a large final compressive stress (t=1200 s), as shown in Figure 14b,d.

**Figure 15:**
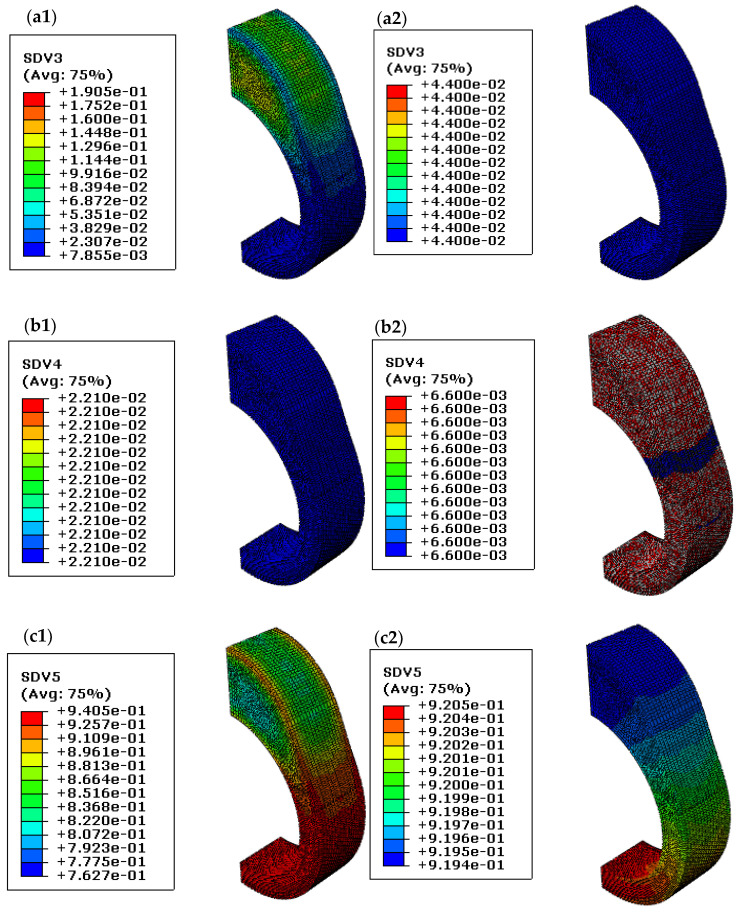
Microstructure distributions under various maximum pearlite volume fractions $f_{3}^{\max}$; (**a1**–**c1**): Pearlite, bainite, and martensite distribution with $f_{3}^{\max}=0.2153$; (**a2**,**b2**,**c2**): Pearlite, bainite, and martensite distribution with $f_{3}^{\max}=0.044$.

To analyze the maximum compressive stress retention, the final microstructure distribution of the quenched cam for the two kinds of different pearlite maximum volume fractions with $f_{3}^{\max}=0.2153$ and $f_{3}^{\max}=0.044$ are shown in Figure 15. During the simulation, the probable maximum phase fractions are extracted from Figure 4b at specific cooling rates. It can be seen from Figure 15a1,c1 that there is an obviously uneven distribution for pearlite (SDV3) and martensite (SDV5) when the maximum pearlite volume equals 0.2153. Since the same heat transfer coefficient curve has been used for the calculation, the major reason for the uneven distribution of pearlite is owing to a high start temperature of bainite (387 °C for $f_{3}^{\max}=0.2153$ and 200 °C for $f_{3}^{\max}=0.044$). In addition, the difference in residual austenite distribution after diffusional transformation affects the uneven distribution of martensite. Conversely, the microstructure distribution of pearlite, bainite (SDV4), and martensite for a maximum pearlite volume that equals 0.044 is all approximately uniform, as presented in Figure 15a2–c2. Therefore, it can be predicted that the uneven microstructure distribution during phase transformation is beneficial for compressive stress retention. Usually, it is difficult to confirm the exact quenching media only according to the cooling rate. Therefore, the residual stress and the final phase volume after quenching under different combinations of probable maximum transformation fractions are hoped to be verified in future work.

## 4. Conclusions

Based on the linear mixture law of the general multiphase material property, a coupled thermomechanical and metallurgical model is successfully developed to calculate the temperature fields, phase volume fractions, stress fields, and hardness in the quenching of the 100Cr6 steel assembled cam via the ABAQUS user subroutines. The influences of different quenchants and probable maximum phase volume fractions on the metallurgical and thermomechanical behaviors are analyzed to investigate the compressive stress retention in the quenched steel. In addition, the experimental tests of surface stress and hardness are carried out to verify the theoretical model. The main conclusions drawn from this paper are summarized as follows:

(1) During the quenching process, the stress tends to be compressive once a phase transformation occurs. Then, the stress releases gradually due to the temperature and microstructure uniformity.
(2) Compared with the thermal stress, the structural stress plays a dominant role in the final surface stress state in the quenched cam. The predicted final compressive residual stress on the cam surface around the buffer section is −63.3 MPa, which is very close to the experimental result −67.8 MPa.
(3) Compared with the conventional quenching oil, the maximum compressive stress, the maximum martensite volume fraction, and the surface hardness of the fast oil quenched cam are larger. The better compressive stress retention of the fast oil quenched cam is owing to the slower stress relief followed by microstructure homogenization. Therefore, a slower martensitic transformation rate is suggested to obtain a quenching compressive stress eventually.
(4) The uneven distributions of microstructures such as pearlite and martensite are beneficial for the compressive stress retention. In addition, a certain amount of martensite volume fraction after quenching is necessary for the final compressive surface stress.

## Figures and Tables

**Table 1:** Phase transformation latent heat of heat-treated 100Cr6 [16,17,21].

**Latent Heat** **(mJ/mm3)**	Austenite to ferrite	3.05 × 10^−4^
	Austenite to pearlite	4.4 × 10^−4^
	Austenite to bainite	4.4 × 10^−4^
	Austenite to martensite	6.48 × 10^−4^
	Original structure to austenite	−3.05 × 10^−4^
**Thermal Expansion Coefficient of Different Phases** **(1/K)**	Austenite	22 × 10^−6^
	Ferrite	16.14 × 10^−6^
	Pearlite	15.3 × 10^−6^
	Bainite	14 × 10^−6^
	Martensite	11.5 × 10^−6^
	Original phase	15 × 10−6
**Volumetric Expansion**	Austenite to ferrite	2.17 × 10^−3^
	Austenite to pearlite	4.81× 10^−3^
	Austenite to bainite	5 × 10^−3^
	Austenite to martensite	7.5 × 10^−3^
	Original structure to austenite	−3 × 10^−3^

**Table 2:** Some material properties of 100Cr6 steel.

Density (kg/m^3^)	Young’s Modulus (GPa)	Poisson’s Ratio	Conductivity (W/m/K)	Specific Heat (J/kg/K)
7.83 × 10^3^	212	0.269	30	700

**Table 3:** Chemical composition of 100Cr6 steel.

Element	Fe	C	Si	Mn	Cr
Mass (%)	96.91	0.99	0.25	0.35	1.5

**Table 4:** Technical parameters for quenching oil.

Model	Viscosity at 40 °C (mm^2^/s)	Viscosity at 100 °C (mm^2^/s)	Flashing point (°C)	Freezing point (°C)
Conventional	15	4	231	−15

**Table 5:** Surface residual stress and retained austenite content test data.

Sample No.	Etch Depth (mm)	Stress Test (MPa)		Retained Austenite Content	Sample No.	Etch Depth (mm)	Stress Test (MPa)	
		1st	2nd	(%)			1st	2nd
1	0.47	−52.9	−56.6	-	16	0.55	−93.8	−84.7
2	0.55	−99.5	−102.8	3.7	17	0.53	−82.4	−74.3
3	0.5	−80.7	−72.7	4.9	18	0.47	−60.6	−54.9
4	0.51	−88.5	−93.1	-	19	0.49	−65.9	−65.4
5	0.47	−42.3	−34.7	6.6	20	0.51	−67.4	−78.5
6	0.56	−93.7	−89.9	-	21	0.55	−104.9	−97.1
7	0.57	−70.7	−66.6	3.8	22	0.58	−73.4	−78.5
8	0.48	−72.4	−53.5	5.7	23	0.51	−66.1	−57.1
9	0.51	−91.5	−91.6	6.5	24	0.5	−53.4	−65.4
10	0.51	−59.6	−76.3	4.1	25	0.51	−68.9	−65.1
11	0.54	−51.9	−56.9	-	26	0.55	−54.5	−62.6
12	0.56	−52	−53.6	2.9	27	0.56	−77.5	−80.3
13	0.51	−65.9	−58.5	-	28	0.47	−41.6	−56.9
14	0.54	−41.5	−50.6	-	29	0.5	−49	−59.5
15	0.54	−46.1	−45	-	30	0.59	−65.1	−67.7

**Table 6:** Surface hardness test data of the quenched sample.

**Group**	**1**	**2**	**3**	**4**	**5**	**6**	**7**	**8**	**9**	**10**
**Hardness (HRC)**	62.8	63	62.9	63.4	63.5	62.9	63.7	63.5	63.1	63.5

**Table 7:** Surface hardness on the buffer section of the tempered cam measured by the Rockwell hardness tester.

**Sampling Number**	**1**	**2**	**3**	**4**	**5**	**6**	**7**	**8**	**Standard**
**Hardness (HRC)**	61.3	61.4	60.9	61	60	59.8	58.8	59.7	54–61

**Table 8:** The microhardness distribution measured by the Vickers hardness tester.

Serial Number	Thickness (μm)	Microhardness (HV)	Transform Values (HRC)	Effective Hardened Layer Thickness with Microhardness 520 HV1 (mm)
1	200	706.33	60.35	1.5–2.5
2	400	701.4	60.14	
3	600	682.03	59.27	
4	800	682.03	59.27	
5	1000	698.95	60.04	
6	1200	706.33	60.35	
7	1400	691.54	59.71	
8	1600	668.12	58.61	
9	1800	656.85	58.08	
10	2000	561.4	53.03	
11	2200	412.44	42.7	

## Data Availability

Not applicable.
